# Intestinal dysbiosis associated with non-nutritive sweeteners intake: an effect without a cause?

**DOI:** 10.3389/fnut.2025.1694264

**Published:** 2025-11-04

**Authors:** Luigi Marongiu, Ewa Brzozowska, Svetlana Hetjens, Ludwig E. Hoelzle, Sascha Venturelli

**Affiliations:** ^1^Department of Nutritional Biochemistry, University of Hohenheim, Stuttgart, Germany; ^2^Laboratory of Medical Microbiology, Hirszfeld Institute of Immunology and Experimental Therapy, Polish Academy of Sciences, Wroclaw, Poland; ^3^Department of Medical Statistics, Biomathematics and Information Processing, University Clinic Mannheim, Mannheim, Germany; ^4^Department of Food Microbiology and Hygiene, Institute of Food Science and Biotechnology, University of Hohenheim, Stuttgart, Germany; ^5^Department of Vegetative and Clinical Physiology, Institute of Physiology, University Hospital Tübingen, Tübingen, Germany; ^6^HoLMiR-Hohenheim Center for Livestock Microbiome Research, University of Hohenheim, Stuttgart, Germany; ^7^Institute of Animal Science, University of Hohenheim, Stuttgart, Germany

**Keywords:** non-nutritive sweeteners, dysbiosis, ace-K, advantame, aspartame, neotame, saccharin, stevia

## Abstract

Non-nutritive sweeteners (NNS) are present in various commercial articles, from foodstuffs to oral hygiene products. Despite their alleged safety, mounting evidence indicates that NNS intake is associated with an alteration of intestinal bacterial populations (dysbiosis) in animals and humans. Since NNS are commercialized based on the assumption that they are not metabolized by human cells and negligible effect on bacterial, the insurgence of dysbiosis associated with NNS intake remains unexplained. The current review aims to assess the effect of selected NNS (acesulfame potassium, advantame, aspartame, neotame, saccharin, stevia, and sucralose) on the human intestinal microbiota. Findings from this review suggests that NNS intake is linked not only to alterations in human physiology but also to modifications of bacterial biochemistry, including the hindrance of quorum sensing pathways, in a species-specific manner. Moreover, there were suggestions that NNS could also affect the biology of phages, namely by binding to the active sites of proteins involved in the infection process and altering the induction rate of prophages. The studies gathered in the present review provide a framework for understanding how NNS might be connected to dysbiosis, both directly through alterations in bacterial biochemistry and indirectly through impaired phage activity.

## Introduction

Currently, obesity affects roughly one in eight people worldwide and, since 1990, the percentage of obese adults and adolescents has doubled and quadrupled, respectively, with over 40% of adults being overweight (1). Obesity and high body weight are linked to various medical conditions, including type 2 diabetes, atherosclerosis, depression, and cancer, with a high burden on people’s quality of life and the health systems around the world (2). To address this ongoing outbreak of weight-related problems, the World Health Organization (WHO) has suggested limiting the amount of free sugar in foodstuffs to 10% the daily energy intake (3). Such a goal was followed to the food industry’s massive employment of non-nutritive sweeteners (NNS, also called non-caloric, high-intensity, or artificial sweeteners) (4).

NNS are so defined because, unlike natural sweeteners such as glucose (dextrose), fructose, or maltose, they do not provide energetic input to human cells (5). The WHO, the American Food and Drug Administration (FDA), and the European Food Safety Authority (EFSA) all declared NNS safe for human consumption (6). However, the food authorities also introduced in 1961 a quantitative evaluator of NNS intake, the acceptable daily intake (ADI), which is defined as the daily amount of a sweetener per body-weight ingestible throughout a person’s lifetime without appreciable health risk (7–11).

NNS are now present in a wide variety of goods, sometimes in the absence of consumer awareness, ranging from soft drinks to cereals, from vitamin supplements to oral hygiene products (12–14). The global yearly consumption of NNS reached 117,000 tons in 2021 (15), and it has been observed that the intake of NNS-containing drinks doubled in children between 2000 and 2008 (16).

The NNS ADI varies according to the sweetener and different countries might adopt distinct ADI levels (17, 18). It has been estimated that one person should drink about 20 cans of diet soda containing aspartame, 800 cans of diet beverages containing saccharin, or 14 servings of iced tea containing sucralose to reach the respective ADI (19, 20). Although such amounts appear unattainable (21–23), it has been reported that the ADI is exceeded in several cases, particularity by youngsters (24–26).

In Germany, where about 89% of soft drinks contain NNS (27), a study carried out on a group of 2,291 individuals assessed that 99.8% of the participant did not exceed ADI intake (25). Nonetheless, extrapolating from these data and considering a German population of 83.5 million (28), one might speculate that 0.2% of individuals who consume NNS above the ADI level would correspond to approximately 1.7 million people at risk of excessive NNS intake. Although the exposure to NNS, particularly stevia, is increasing worldwide, especially due to consumption of soft drinks, there is a paucity of recent data monitoring the actual consumption of NNS in the German population (29–31).

Recently, the WHO carried out a systematic re-evaluation of clinical trials on physiological markers, finding that not only does NNS intake not reduce body fat, as previously claimed, but it could also increase the risk of type 2 diabetes, cardiovascular disease, and mortality in consumers (32). Thus, the WHO has revised its stance on NNS safety and now recommends avoiding NNS consumption to control body weight (33, 34). Such re-evaluation highlights the necessity of understanding the effect of NNS intake on human physiology. The data derived from clinical trials regarding the effect of NNS intake on human subjects is controversial, and, in general, the long-term effect of NNS on human health needs to be more adequately investigated (35), especially considering that more people are exposed to NNS than those who knowingly consume them. For instance, an NNS intervention study reported that eight (44.4%) of 18 healthy participants chosen as baseline non-NNS consumers showed sucralose in their urine (average concentration 0.6 mM) even before the actual trial begun (36).

Each consumer can excrete tens to hundreds of NNS milligrams per day in the urine (37–39) and NNS have also been detected in amniotic fluids at concentrations of nanograms per milliliter (40). Due to their chemical stability, NNS are not removed during wastewater treatment; thus, they can reach concentrations of about 2.5 mg/L in effluvial water and have become a widespread environmental contaminant that is employed as trackers of human pollution (41–44). For example, environmental exposure to ace-K has been linked to an increased cellular damage in carp (45). Contaminated water and soil carry the risk of transferring NNS back into the food chain, with the potential of indirect NNS exposure through environmental contamination (46).

Moreover, the scientific assessment of NNS safety is more demanding than it might appear. For instance, several NNS are often mixed in foodstuffs, making it more challenging to assess the individual roles of NNS on human physiology (47). In particular, while dysbiosis is a common outcome associated with NNS intake, its effects are highly variable, depending on individual dietary habits and the genetic backgrounds of both the host and microbes, suggesting a personalized approach for further investigation in this area of nutrition (48, 49). Furthermore, the effects of NNS on human physiology are typical of prolonged contact with low concentrations, a combination that is difficult to replicate experimentally (50). In addition, the type of study can also determine a substantial bias in the results. For instance, it has been suggested that trials funded by sweetener manufacturers tended to report a lower NNS (namely, aspartame) risk association than independent studies (51).

Nonetheless, a growing number of reports suggest that NNS consumption can be associated with adverse physiological effects, particularly due to alterations in the intestinal microbiome compared to normal conditions (dysbiosis). A cross-sectional observational study of human volunteers showed a negative correlation between NNS intake and the abundance of different types of bacteria, including butyric acid-producing ones, in the colon (52). It has been shown that ace-K, aspartame, saccharin, and sucralose increased the horizontal gene transfer among bacteria, both at the intra-species (among *Acinetobacter baylyi* strains) and the inter-species (between *A. baylyi* and *Bacillus subtilis*) levels (53). Remarkably, it has also been shown that these NNS added at a concentration of 1–5 mM for 24 h decreased the growth of the acidogenic *Streptococcus mutans* but left unaffected that of the alkali-producing *Streptococcus sanguis* (54). Based on a survey of 28 clinical studies, there was a tendency for an increased abundance of members of the *Enterobacteriaceae* family and decreased abundance of members of the *Clostridium* cluster XIVa in NNS consumers compared to healthy controls (55). In addition, bacteria extracted from 13 healthy volunteers and exposed to sucralose showed an increased abundance of *Escherichia* and *Shigella* genera, whereas aspartame increased the abundance of bifidobacteria; the production of short-chain fatty acids (SCFA) was also altered by these sweeteners (56). Sucralose has been indicated as a possible cause of the insurgence of inflammatory bowel disease (57). If NNS can affect some bacterial species or even strain differently from others, it can be speculated that NNS intake could raise the risk for an imbalance in the microbial growth that could explain the observed dysbiosis in NNS consumers.

The goal of this review was to determine the direct effects of popular NNS on gut microbes to identify the mechanism underlying the development of dysbiosis associated with the consumption of sweeteners. The most prevalent NNS include acesulfame potassium (ace-K), advantame, aspartame, neotame, saccharin, stevia, and sucralose (4, 58, 59). This review will focus on the direct interaction between NNS and bacteria, based on *in vivo* and *ex vivo* trials in animals, as well as *in vitro* experiments. The review will also examine the potential impact of NNS on phages, considering the crucial role these microbes play in shaping the intestinal microbiome.

The articles presented in the present review were obtained primarily by searching the National Library of Medicine (NLM) with the *PubMed* tool (60) with the keywords: *((((((Inflammation[MeSH Terms]) OR (Inflammation)) OR ((Dysbiosis[MeSH Terms]) OR (Dysbiosis))) OR (Pathological Conditions, Signs and Symptoms[MeSH Terms])) OR ((obesity) OR (obesity[MeSH Terms]))) OR ((Gastrointestinal Microbiome[MeSH Terms]) OR (Gastrointestinal Microbiome))) AND ((Non-Nutritive Sweeteners[MeSH Terms]) OR (Non-Nutritive Sweeteners))*. Furthermore, the NLM’s database of clinical trials (61) was searched using the following keywords: *Condition/disease: Non-Nutritive Sweeteners; Other terms: Inflammation OR obesity OR microbiome*. Both queries were initiated on November 15, 2022. Relevant papers were included based on their titles and abstracts. Additional literature was obtained by further exploring works related to the selected studies.

## NNS structure and metabolism

### Ace-K

Ace-K is an oxathiazinone dioxide salt that is efficiently absorbed by the small intestine without metabolization. Thus, ace-K is rapidly transported into the bloodstream and then eliminated through the urine, although 1% of the ingested dose is released into the feces (62). In pregnant women, ace-K has been recovered in amniotic fluid (40) and is transmitted to newborns through breastfeeding (63). However, there are no reports of adverse effects in children. The by-products of ace-K are potassium and acetoacetamide, and dietary NNS generates concentrations of these metabolites that are considered well within the safety levels (64, 65). Ace-K was cleared for human consumption in the 1970s, albeit the clinical testing was later deemed unsatisfactory and additional trials were never performed (66). Moreover, it has been shown that both mouse adipocytes 3 T3-L1 and human primary mesenchymal stem cells exposed to ace-K exhibited enhanced adipogenesis (67).

**Figure 1:**
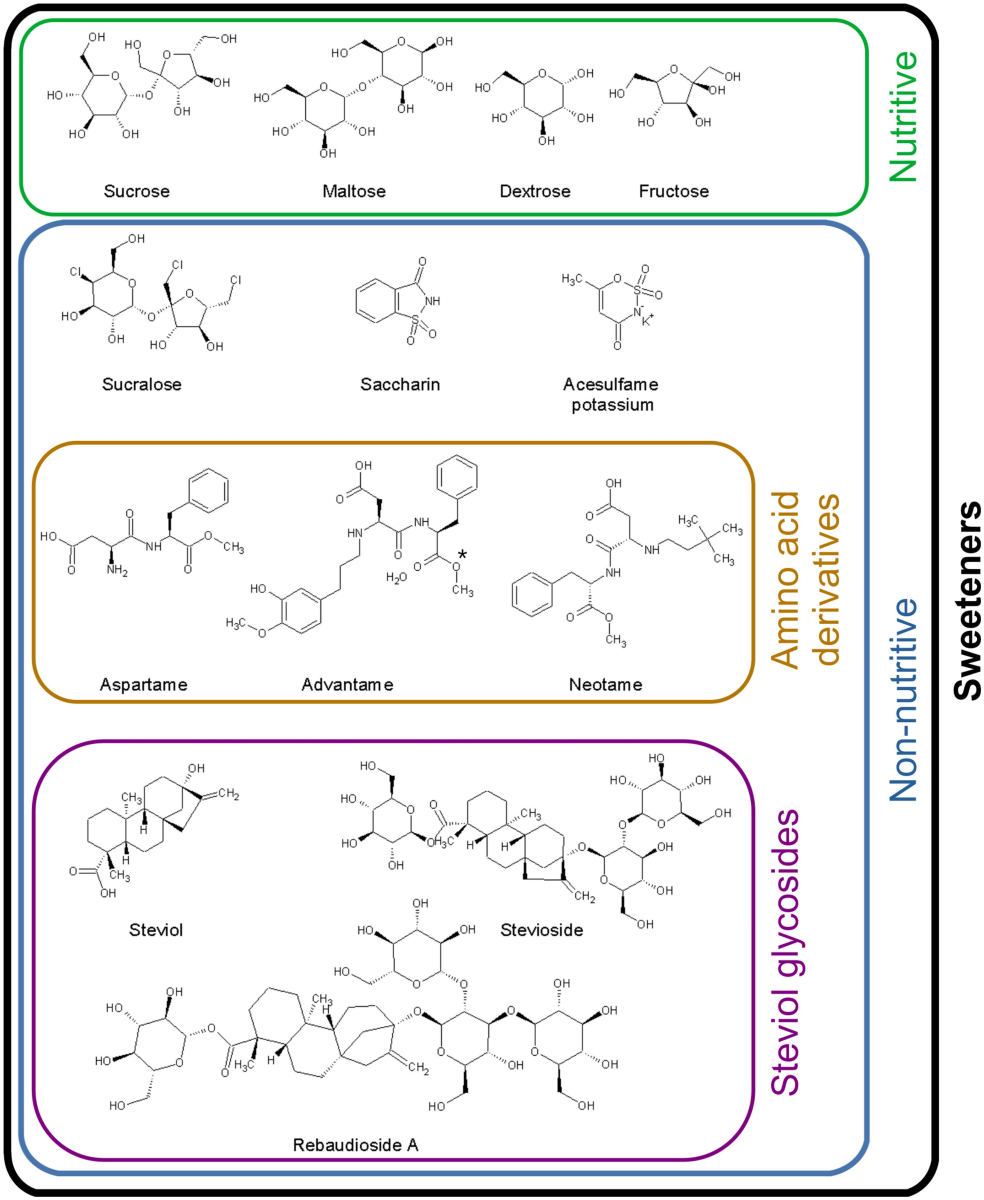
Chemical structure of the sweeteners included in the present review. Comparison of the structures of natural and non-nutritive sweeteners. Dextrose (also known as glucose) and fructose are the basic units of monosaccharides; maltose and sucrose are disaccharides. Sucrose is the reference molecule for the sweetening power of sweeteners. The de-esterification of advantame (*) generates ANS9801-acid.

It has also been reported that several environmental bacteria (belonging to the families *Boseaceae*, *Bradyrhizobiaceae*, *Chelatococcaceae*, *Methylophilaceae*, *Phyllobacteriaceae*, and *Pseudomonadaceae*) can metabolize this NNS using it as an energetic source (68, 69). The biodegradation of ace-K to sulfamic acid has been reported in lakes, rivers, and wastewater treatment plants, but only in aerobic conditions (70, 71). Since the gastrointestinal tract (GIT) is virtually anaerobic (72), the fate of this NNS in the GIT remains largely unknown (see Figure 1).

### Aspartame, advantame, and neotame

Aspartame, formally known as aspartyl-phenylalanine methyl ester (or (N-L-*α*-aspartyl)-L-phenylalanine methyl ester), is a methylated dipeptide of the amino acids L-aspartic acid and L-phenylalanine. Advantame (N-[N-[3-(3-hydroxy-4-methoxyphenyl)propyl]-α-aspartyl]-L-phenylalanine 1-methyl ester) and neotame (N-[N-(3,3-dimethylbutyl)-1-α-aspartyl]-L-phenylalanine 1-methyl ester) are aspartame derivatives (73). Compared to aspartame, the former (developed through computer-based design by the sweetener producer firm *Ajinomoto* in 1987) has higher thermostability (74); the latter, released in 1991, has higher water solubility. The International Agency for Research on Cancer (IARC) has registered aspartame as a possible carcinogenic agent for humans (75).

Aspartic acid and phenylalanine are released, together with methanol, from the matabolization of aspartame by peptidases and esterases present in the intestinal cells (62). The hydrolysis of advantame in the intestinal tract also produces methanol as well as de-esterified advantame (also known as ANS9801-acid) (76, 77). The latter molecule is further metabolized to N-(3-(3-hydroxy-4-methoxyphenyl))-propyl-L-aspartic acid (HF-1) (78). The hydrolysis of neotame produces dimethylbutylaspartylphenylalanine (DMB-Asp-Phe) and methanol (73).

Inside the enterocytes, aspartic acid is converted to oxalacetate, which is involved in gluconeogenesis; phenylalanine is transformed to tyrosine, whereas methanol is converted into formaldehyde and, in turn, to formic acid and carbon dioxide. The excess of these metabolites, the same as those obtained from the digestion of natural food, is excreted in the urine. Although methanol is toxic, the levels generated by the digestion of aspartame are considered well below the safety threshold (79). No aspartame has been recovered during breastfeeding (80). Aspartame-derived metabolites are readily adsorbed by the small intestine and delivered by the portal vein to the liver; therefore, it does not reach the colon, whereas both advantame and neotame metabolites were recovered from feces and urine (73).

High levels of aspartic acid or phenylalanine may cause health issues, but dietary amounts of aspartame are considered to produce negligible amounts of these metabolites (80). However, people with phenylketonuria should avoid ingesting aspartame because they lack a functional phenylalanine hydroxylase and, therefore, cannot convert phenylalanine to tyrosine (81). High levels of phenylalanine have been linked to an increased risk of seizures (82).

### Saccharin

Saccharin is a benzoic acid sulfimide that is moderately absorbed by the small intestine: while about 85–95% of it is delivered to the bloodstream and excreted in the urine, the rest reaches the colon unaltered (62). Saccharin is not metabolized by animal cells (83), indicating physiological inertness in humans, which has led to its widespread use in food products.

Experimental models performed in the 1970s-1980s reported an increased risk of bladder cancer in rodents fed with high-dose saccharin, leading to a legal requirement to display a warning label on saccharin-containing items (51). However, such a requirement was removed in 2000 based on the objection that rodents do not constitute a proper model for human physiology and that the concentrations utilized in early experiments were well above the ADI (84). Nonetheless, it was later demonstrated that human and mouse adipocytes treated with saccharin exhibited an abnormally activated Akt signaling pathway, leading to enhanced adipogenesis and reduced lipolysis (67).

### Stevia

Stevia is a generic term for the extract of the shrub *Stevia rebaudiana* (fam. *Asteraceae*), also known as candyleaf, which is widespread in south America (85). Stevia has been utilized by indigenous tribes for centuries, but it was not officially recognized by Western scientists until 1887. It was introduced to the Japanese food industry in the 1970s, received approval from the FDA in the 1990s, and was introduced in the European market in 2011 (86, 87).

In 1931, the active factor of candyleaf extracts was described to contain a mixture of glycosides, the most abundant being steviol, stevioside, rebaudioside (reb) A-F, dulcoside, and steviolbioside (86, 88). The steviol glycosides share a diterpene steviol moiety (89). The term ‘stevia’ will be used in this review as a synonym for steviol glycosides to indicate that no active factor has explicitly been disclosed in the cited literature.

Stevia reaches the colon unaltered because animal cells are unable to metabolize its glycosides (62). Intestinal bacteria, specifically members of the genus *Bacteroides*, have been shown to metabolize steviol glycosides, producing steviol as the final product, which is resistant to further degradation (90, 91). Steviol is then absorbed by the colonic epithelium and released into the bloodstream (89). Interestingly, the microbial enzyme involved in the degradation of steviosides, sennoside hydrolyzing glycosidase, is usually inhibited by glucose (92); thus, it might be expected that its expression would be increased in diets that are poor in this natural saccharine (93).

### Sucralose

Sucralose is a chlorinated disaccharide obtained by exchanging three hydroxyl groups of sucrose with chlorine. It is poorly absorbed by the small intestine so that about 85% of it reaches the colon and is excreted in the feces (62, 94). It is commonly assumed that sucralose is metabolized neither by animal nor by bacterial enzymes (95), a feature that advocated for its physiological safety. Nonetheless, it has been reported that environmental bacteria can degrade this NNS, although without a substantial energetic gain, to 1,6-dichloro-1,6-dideoxy-D-fructose and uronic acid (96, 97).

## NNS impact on bacteria

### Overview

In the following sections, experimental studies investigating the direct effect of NNS on the biology of bacteria will be discussed. The results are summarized in Table 1; selected characteristics of NNS are reported in Table 2.

**Table 1:** Effects of non-nutritive sweeteners on bacteria^a^.

Effect	Ace-K	A.A.N.^b^	Saccharin	Stevia	Sucralose	Ref.
Bacteriostasis						
*A. viscosus*	NC	NC	NC	NC	+(13.9–55.5)	(118)
*E. faecalis* 19433	NC	–(0.1)	–(0.1)^c^	NC	–(0.1)	(107)
*E. coli* 10418	NC	–(0.1)	±(0.1)^c^^,^^d^	NC	–(0.1)	(107)
*E. coli* DSM 613	NC	NC	NC	±(0–520)^d^	NC	(112)
*E. coli* DSM 5695	NC	NC	NC	±(0–520)^c^^,^^d^	NC	(112)
*E. coli* HB101	+(124)	NC	+(136.5)	+(25.9)	+(62.9–125.7)	(98)
*E. coli* K-12	±(124)	NC	+(136.5)	–(25.9)	+(62.9–125.7)	(98)
*E. coli* K802NR	–(5.0)	±(1.4)	+(2.7)	NC	+(25.2)	(101)
*L. amylovorus*	NC	NC	–(0.8)	NC	NC	(110)
*P. syringae* DSM 21482	NC	NC	NC	±(0–520)^c^^,^^d^	NC	(112)
*S. mutans*	NC	–(0.07–68)	+(0.1–110)	NC	NC	(108)
*Streptococcus* spp.	NC	NC	NC	NC	+(13.9–55.5)	(118)
Dysbiosis	+(29.8–124)	+(20.4–84.9)	+(136.5)	+(1–25.9)	+(15.1–125.7)	(93, 98, 99, 104, 105, 109, 114, 121, 202)
Alteration in metabolism	+(29.8)	+(20.4)	+(140)	+(NA)	+(15.1)	(99, 102, 114)
Increased mutation rate	NC	NC	NC	NC	+(157.2)	(119)
Genotoxicity						
*E. coli* DPD2794	+(49.7)	+(13.6)	+(27.3)	NC	NC	(103)
*E. coli* TV1061	–(NA)	–(NA)	+(27.3)	NC	NC	(103)
*E. coli* DPD2544	–(NA)	–(NA)	–(NA)	NC	NC	(103)
Increased recombination						
Intra-species	+(1.5 × 10^−4^ –1.5)	+(1.0 × 10^−4^–1.0)	–(1.6 × 10^−4^–1.6)	NC	+(8.0 × 10^−5^–8.0 × 10^−1^)	(15, 102)
Inter-species	+(1.5 × 10^−4^ –1.5)	+(1.0 × 10^−4^ –1.0)	+(1.6 × 10^−4^ –1.6)	NC	+(8.0 × 10^−5^–8.0 × 10^−1^)	(15, 102)
Biofilm formation promotion						
*E. coli*	NC	+(1.0 × 10^−4^ –0.1)	+(1.6 × 10^−4^ –0.1)	NC	+(8.0 × 10^−5^–0.1)	(15, 107)
*E. faecalis*	NC	+(0.1)	–(0.1)	NC	–(0.1)	(107)
Enhanced cytotoxin production	NC	+(0.1)	–(0.1)	NC	+(0.1)	(107)
QS impairment	–(5.0)	±(1.4)^e^	+(2.7)	NC	+(25.2)	(101)

a +: effect observed experimentally; −: effect not observed experimentally; ±: conflicting results; NA: value not available; NC: experiment not conducted. Exposure concentrations are given in mM.

b Aspartame, advantame, or neotame.

c Based on exposure to whole stevia extracts.

d Concentration estimated from the molecular weight of rebaudioside A (967.0).

e Positive effect with aspartame; negative effect with advantame/neotame.

**Table 2:** Characteristics of selected non-nutritive sweeteners.

NNS	Molecular weight (g/mol)	AC^a^	Discovered/approved	SP^b^	ADI^c^	Fraction reaching colon (%)	Concentration in water^d^	Chemical class^e^	Final metabolic products
Ace-K	201.2	E950	1967/1984	150–200	9/15	1	49.7 pM- 2.8 nM	Sulfuric acid derivative	Acetoacetamide, potassium
Advantame	476.5	E969	1987/2014	37,000	5/33	77–96	–	Dipeptide	ANS9801, methanol
Aspartame	294.3	E951	1965/1984	200	40/50	0	34.0 pM	Dipeptide	Aspartic acid, phenylalanine, methanol
Neotame	378.5	E961	1991/2008	7,000–13,000	2/0.3	50^f^	–	Dipeptide	DMB-Asp-Phe, methanol
Saccharin	183.2	E954	1878/1977	240–300	5/15	5–15	54.6 pM- 1.7 nM	Benzisothiazole derivative	None
Steviol glycosides^g^	≥ 318.4^h^	E960	1887^i^/2008	300	6–16/4	100	–	Diterpenoid derivatives	Steviol
Sucralose	397.6	E955	1976/2000	750	15/5	85	25.2 pM- 2.4 nM	Disaccharide	None

a Additive code.

b Sweetening power compared to sucrose (30 g/L at 20 °C).

c Admissible daily intake in the EU/USA (mg/kg/day).

d Derived from Praveena et al. (42); comprises tap, surface, ground, sea, and lake water in Europe.

e Derived from PubChem Kim et al. (203).

f As as 3,3-dimethylbutylaspartylphenylalanine (DMB-Asp-Phe).

g Component of stevia.

h MW calculated for steviol.

i In use for more than 1,500 years but scientifically described in this year.

### Ace-K

Ace-K had a strain-specific bacteriostatic effect on *E. coli in vitro*: Luria-Bertani (LB) agar supplemented with 2.5% w/v (124 mM) ace-K reduced the number of colony-forming units (CFU) by 90% for *E. coli* strain HB101, and 98% for K-12 (98). Other studies reported a boost in the growth of *E. coli* upon exposure to 6 mg/mL of ace-K, an effect associated with alterations in the bacterial metabolism (99). Others, however, did not report substantial differences in bacterial growth: mice fed ace-K supplements within the ADI for 8 weeks did not show a difference in microbial density or cecal butyrate concentration compared to a placebo group (100). The growth of *E. coli* strain K802NR was not affected by exposure to ace-K at a concentration of 5 mM (101). Bacteria isolated from rat guts and exposed to ace-K displayed inhibited glucose fermentation (102).

Ace-K induced an anti-genotoxic response in *E. coli* strain DPD2794 but not in strains TV1061 and DPD2544 (103). Ace-K at a concentration of at least 0.03 mg/L increased over three times the recombination frequency between *E. coli* K-12 strains and four times between *E. coli* and *Pseudomonas alloputida* (15). The authors of such a study also reported that the high recombination rate was decreased upon treatment with radical scavengers, suggesting that reactive oxygen species (ROS) were involved in the process. Membrane permeability was also increased upon treatment with ace-K (15). Because the concentration in the experiment was lower than the concentration of NNS in the urine, the authors argued that ace-K could increase the rate of horizontal gene transfer in the human gut, with a higher risk of spreading antibiotic resistance genes (15).

### Aspartame, advantame, and neotame

Dysbiosis was observed in newborn mice breastfed by mothers provided with chow supplemented with aspartame, correspondent to the human ADI, in particular, with higher production of propionate and butyrate and decreased lactose fermentation (104). Mice fed with a concentration within the ADI for aspartame over a period of 8 weeks showed impairment of glucose tolerance and higher abundance of members of the family *Enterobacteriaceae* as well as *Clostridium leptum*, an SCFA producer compared to controls (105, 106).

Aspartame was not bacteriostatic against either *E. coli* 10,418 or *Streptococcus mutans* 19,433, but it increased biofilm formation in *E. coli* and *Enterococcus faecalis* as well as enhanced their adhesion to Caco-2 intestinal cells (107, 108). Similarly, advantame (0.4 mM) and neotame (0.5 mM) did not affect the growth of *E. coli* K802NR whereas aspartame (1.4 mM) showed bacteriostatic activity (101). However, other studies did report a bacteriostatic effect of aspartame (20.4 mM) on *E. coli* along with altered fatty acid metabolism (99). Moreover, aspartame stimulated the production of cytotoxins in *E. faecalis*, with subsequent reduced viability of Caco-2 cells (107). Aspartame induced DNA damage in *E. coli* strain DPD2794 but not in strains TV1061 and DPD2544 (103). Molecular docking studies showed that aspartame could bind the hydrophobic pocket involved in the detection of the quorum sensing (QS) modulator 3-oxo-C12-HSL (LasR) of *Pseudonomas aeruginosa*, in particular by establishing connections with residue Val_76_, with an overall affinity of −8.6 kcal/mol, impairing the quorum sensing pathway of this bacterium (101).

Aspartame (0.1 μM) increased four-fold the recombination frequency among *E. coli* K-12 strains as well as inter-species recombination (*E. coli* to *P. alloputida*) (15). The increased recombination rate was associated with a higher ROS concentration in the bacterial cells and higher membrane permeability (15).

Models based on CD-1 mice fed for 4 weeks with neotame at a concentration equivalent to 2.5 times the human ADI showed alterations of the enteric microbiome (109). The alterations in treated mice compared to controls included enrichment of members of the genus Bacteroides (particularly those belonging to the family S24-7) and depletion of members of the families *Lachnospiraceae* and *Ruminococcaceae*. Such a modification of the microbiota was also associated with a shift in bacterial biochemistry, characterized by a reduction in the concentrations of malic acid, mannose-6-phosphate, and glyceric acid, among others. On the other hand, there was an increase in lipids such as linoleic and stearic acids.

### Saccharin

Saccharin at a concentration of 2.7 mM inhibited the growth of *E. coli* strain K802NR (101) and exhibited bacteriostatic effect on *E. coli* strain 10,418 at a concentration of 1 mM but not at 0.1 mM, although it did not affect the growth of *E. faecalis* (107). Saccharin displayed a species-specific bacteriostatic effect on *E. coli*: LB agar supplemented with 2.5% w/v (137 mM) saccharin reduced the number of CFU by 90% for strain HB101, and almost 100% for K-12 (98). Studies in rats reported discordant results. It was shown that saccharin did not affect the growth of *Lactobacillus amylovorus* strain 4,228 (110). In contrast, others have shown that saccharin inhibits the fermentation of glucose in the gut (102). Saccharin at a concentration of 0.1 μM did not affect the recombination frequency between *E. coli* strains or between *E. coli* and *P. alloputida* (15).

*Streptococcus mutans* was inhibited in a dose-dependent manner by saccharin (108). Saccharin provided at a concentration corresponding to the ADI increased biofilm formation and cellular adhesion to Caco-2 cells in *E. coli* and *Enterococcus faecalis* cultures (107). Furthermore, saccharin significantly increased the invasion index of *E. faecalis* but not that of *E. coli*; however, *E. coli* exposed to saccharin increased the production of cytotoxins reducing the viability of Caco-2 cells (107). Saccharin induced chromosomal damage in *E. coli* strains DPD2794 and TV1061 but not in strain DPD2544 (103).

Like aspartame, saccharin was shown by molecular docking to bind the QS receptor LasR, in particularly by binding to Val_76_, with an affinity of −7.3 kcal/mol; thus, impairing *P. aeruginosa* QS pattern and its inhibiting the bacterial growth and motility (101).

Saccharin also increased tissue inflammation. C57BL/6 J mice fed with saccharin at a concentration of 0.3 mg/mL for 6 months displayed a higher expression of pro-inflammatory markers such as inducible nitric-oxide synthase (iNOS) and TNF-*α*, as well as a higher abundance of some genera such as *Corynebacterium* and *Roseburia*, and a lower abundance of *Ruminococcus* compared to controls (111).

### Stevia

Stevia extracts elicited concentration-dependent species-specific responses in selected bacteria (112). For instance, a 1.5% w/v solution of methanol-extracted stevia caused a significant decrease in the growth of *E. coli* strains 613 and 5,695 but not in *Pseudomonas syringae* strain DSM 21482. However, at a concentration of 3.1%, stevia extracts exhibited a significant bacteriostatic effect on *P. syringae* 21,482, but did not alter the growth of the *E. coli* strains. Instead, at a stevia concentration of 6.2%, *E. coli* 5,695 showed a significant growth increase over the control, whereas *E. coli* 613 and *P. syringae* 21,482 remained unaffected.

It has been reported that rebA could be metabolized by selected members of the genera *Bifidumbacterium* and *Lactobacillus* in a strain-specific fashion: *B. breve* CCDM 562, *B. bifidum* CCDM 559, *B. adolescentis* AVNB3-P1, and *L. mucosae* SP1TA2-P1 showed faster growth than a panel of eleven other strains (113). While the increase in growth rate was deemed too small to provide a significant advantage to these strains, newborn mice fed with stevia showed an increased abundance of propionate- and butyrate-producing bacteria and a decreased abundance of lactose fermenters compared to controls, leading to increased body weight and fat accumulation (104).

RebA displayed a strain-specific bacteriostatic effect *in vitro* on *E. coli* HB101 but not on K-12 (98). Exposure to steviol, a compound produced by bacteria harvested from the colon of volunteers, resulted in a tenfold reduction in propionate production and a change in pH associated with a higher density of bifidobacteria (114). It has been shown that, compared to glucose, stevioside is an inhibitor of anaerobic bacteria, whereas RebA is an inhibitor of aerobic bacteria (93).

Not all effects associated with stevia are adverse. It has been demonstrated that stevia stimulates the expression of sodium/glucose cotransporter 1 (SGLT1) on the surface of rabbit intestinal cells, alleviating the pathogenic symptoms of experimental *E. coli* infection (115). SGLT1, which is activated by glucose, facilitates the absorption of water and other electrolytes into the cell, thereby counteracting the effects of colitis (116). SGLT1 expression is induced by the hormone glucagon-like peptide 2 (GLP-2), released by enteroendocrine cells of the intestine in response to glucose intake (117). Stevia can also activate the excretion of GLP-2 from the enteroendocrine cells by binding to the taste family 1 receptor (T1R) present on the surface of the intestinal epithelial cells (115). The physiological consequences of such an alteration are unknown.

### Sucralose

Sucralose showed a bacteriostatic effect on *E. coli* HB101 and K-12 *in vitro*: LB agar supplemented with 2.5% w/v (63 mM) sucralose decreased the bacterial density by 74%; moreover, the size of the colonies also showed a dose-dependent reduction related to the content of sucralose in the culture medium (98). Similarly, 25 mM sucralose significantly reduced the growth of *E. coli* strain K802NR (101). A slight bacteriostatic effect upon *E. coli* was confirmed at a concentration of 15.1 mM (99). However, *E. coli* strain 10,418 was not affected by sucralose (107). Sucralose at a concentration of 126 mM inhibited the growth of *Streptococcus sobrinus*, *S. sanguis*, *S. challis*, *S. salivarius*, and *Actinomyces viscosus*, all of which are commonly found in the oral microbiome, without entering the bacterial cells (118). Sucralose at a concentration of at least 27.8 mM inhibited the growth of a panel of environmental bacteria (50). Even in this case, the inhibitory effect was obtained without the transportation of sucralose into the bacterial cell. Sub-inhibitory concentrations of sucralose, however, increased the survival rate of *E. coli* BW25113 when exposed to the antibiotic moxifloxacin and enhanced the mutation rate of this strain (119).

Mice fed a supplement of sucralose showed increased body weight and a higher abundance of Bacillota compared to controls (98). Sucralose within the ADI increased biofilm formation and cellular adhesion to Caco-2 cells in *E. coli* and *E. faecalis* cultures (107). Additionally, sucralose enhanced the expression of cytotoxins in both *E. coli* and *E. faecalis*, thereby reducing the viability of Caco-2 cells (107). Maternal sucralose in mice also down-regulated the expression of mucin type 2 and tight junction protein ZO-1, while boosting pro-inflammatory cytokines such as IL-1β and IFN-*γ*, suggesting a morphological alteration of the intestinal epithelium associated with local inflammation (120).

Remarkably, different concentrations of sucralose were linked to dinstinct dysbiotic profiles. Sucralose administered to rats at 0.54 mM increased Bacillota abundance while decreasing the abundance of *Bacteroidetes*, whereas sucralose at 0.78 mM had the opposite effect (121). Nonetheless, both concentrations reduced the abundance of members of the commensal families *Lactobacillaceae* and *Akkermansiaceae*.

Sucralose was also involved in generating an inflammatory micro-environment. C57BL/6 mice fed sucralose at a concentration of 0.3 mM for 6 months exhibited a higher expression of pro-inflammatory markers, such as matrix metalloproteinase 2 and iNOS, along with altered expression of amino acid metabolism and modifications in the microbiome relative abundances (122).

It has been reported that sucralose administered within the ADI to mice for 8 weeks resulted in a dose-dependent reduction in the abundance of bacteria of the *Clostridium* cluster XIVa group and a decrease in the amount of cecal butyrate (100). Sucralose binds to the *P. aeruginosa* QS receptor LasR, particularly by forming a connection with residue Val_76_, with an affinity of −6.1 kcal/mol, thereby inhibiting the growth and motility of this bacterium through impairment of the quorum sensing pathway (101).

Sucralose at a concentration of 0.1 μM was sufficient to promote intra-species recombination in *E. coli* K-12, and inter-species recombination between *E. coli* and *P. alloputida*, with rate increases of 1.5 and 2.6 times over controls, respectively (15). Sucralose treatment increased the production of ROS in the bacterial cells and the permeability of the cells (15).

## NNS impact on bacteriophages

### Overview

Bacteriophages (phages for short) represent a major modulator of bacterial communities (123, 124). Lytic phages provide one level of regulation (‘Kill-the-Winner’ model) by lysing the more abundant species in the community and allowing the proliferation of less competitive bacteria (125). Recent data have shed light on the crucial role of phages in modulating the development and response of the immune system (126–129). To infect their hosts, phages require not only receptors to recognize bacterial surface receptors, such as proteins and carbohydrates present in capsule and cell wall compounds, but also enzymes that can digest the polysaccharides present not only in these structures but also in biofilms’ extracellular matrices (130). For example, the tail tubular proteins TTPAgp31 (gp31 for short) of *Klebsiella pneumoniae* phage KP32 possess glycolytic activity, which enables the virus to diffuse within biofilms (131–133). Nonetheless, it is assumed that most phages in the human GIT are temperate (134). The theoretical frameworks (such as the ‘Piggyback-the-Winner’ and ‘community shuffling’ models) predict that prophage induction at high host densities is a key aspect to stabilize dominant bacterial species and promote diversity through genetic transfer (135–139).

Despite the momentous role that phages play in shaping the intestinal microbiome, the effect of NNS on phage biology has mostly gone overlooked. Due to the lack of experimental data on the impact of non-nutritive sweeteners (NNS) on phage biology, the following sections will investigate how substances like sucrose and polyethylene glycol affect phage particles and their infectivity. These molecules were chosen because they share similar chemical properties with NNS and carbohydrates.

### Phage stabilization

While the literature on how NNS affect the morphology of phages is sparse, it is crucial to recognize that carbohydrates and other compounds significantly influence virion structure. These studies primarily focus on the need to enhance virion stability during industrial storage. In particular, lyophilization is a necessary step in the long-term preservation of viruses and the delivery of phage preparations through spraying; however, it can cause disruptions to virions, resulting in the loss of infectivity (140). Carbohydrates such as lactose, mannitol, polyethylene glycol (PEG), and trehalose are known to prevent virion disruption during lyophilization by forming a protective matrix around the virus shell (141–143). Several sweeteners (dextran, glucose, sucrose, trehalose, mannitol, and xylitol) have been investigated for their properties in protecting phage particles during phage preparation, with 10% w/v sucrose (292 mM) being the most effective (144). Other studies confirmed the protective power of sucralose, applied at a concentration of 2% (58 mM) (145).

By way of example, sucrose is routinely used to stabilize phage particles in lyophilized phage preparations (146). In a process known as “preferential exclusion,” disaccharides can surround a capsid, trapping a layer of liquid water around the virion and protecting it from structural deformation caused by freezing (147). Dextran can protect the capsid from osmotic and heat shocks (148). Steviol glycosides are known, apart from their sweetness, for their emulsification power and are employed to improve food texture (149–151). It has been shown that rebA can form apolar bonds with proteins, such as those found in soy extracts, thereby improving the emulsification of the matrix (152). Remarkably, it has been reported that emulsifiers can alter the intestinal microbiome (153). Nonetheless, the role of rebA in particular and steviol glycosides in general in modulating the homeostasis of the GIT remains poorly characterized.

The concentration of these protective molecules is an important factor to consider. Sucrose at a low concentration (100 mM) showed protective activity against *E. coli* phage CA933P during the lyophilization process (which includes both freezing and drying steps), whereas higher concentrations increased virion disruption (154).

### Alteration of infectivity

There is very limited information regarding the role of NNS on phage infectivity. One study demonstrated that stevia extracts can either enhance or reduce the infectivity of selected phages (112). In particular, methanol-derived stevia extracts exhibited not only different activities against various viruses but also a concentration-dependent behavior. For instance, a concentration of 50% w/v of stevia significantly increased phage MS2 (host: *E. coli* DSM 5695) and T4 (host: *E. coli* DSM 613) densities in comparison with unexposed controls but not that of phage Φ6 (host: *Pseudomonas syringae* DSM 21482). Conversely, 1.5% stevia significantly decreased MS2 and T4 densities compared to unexposed controls, but exposure to 3% solution increased the amount of phages; Φ6 had the opposite trend (112).

Phage infection is affected by its environment. For instance, the infectivity of phage lambda towards *E. coli* was decreased in the presence of lactose, possibly due to this carbohydrate hindering the adsorption step (155).

Only one study analyzed the direct interaction between sweeteners, albeit natural, and phage proteins involved in the infection process (132). Docking analysis demonstrated that maltose could fit into a pocket within gp31, establishing a hydrogen bond with residues Asp_131_, Asp_133_, and Glu_134_, with Asp_133_ being part of the catalytic site (132). Consequently, it was hypothesized that the binding side of gp31 would accommodate disaccharides because larger molecules would cause the protein to unfold. The authors of that study also noted that the binding to maltose was not very specific, implying that gp31 might bind to various saccharides. Such data can lead to speculation that NNS might have the potential to bind gp31 or other phage proteins.

**Figure 2:**
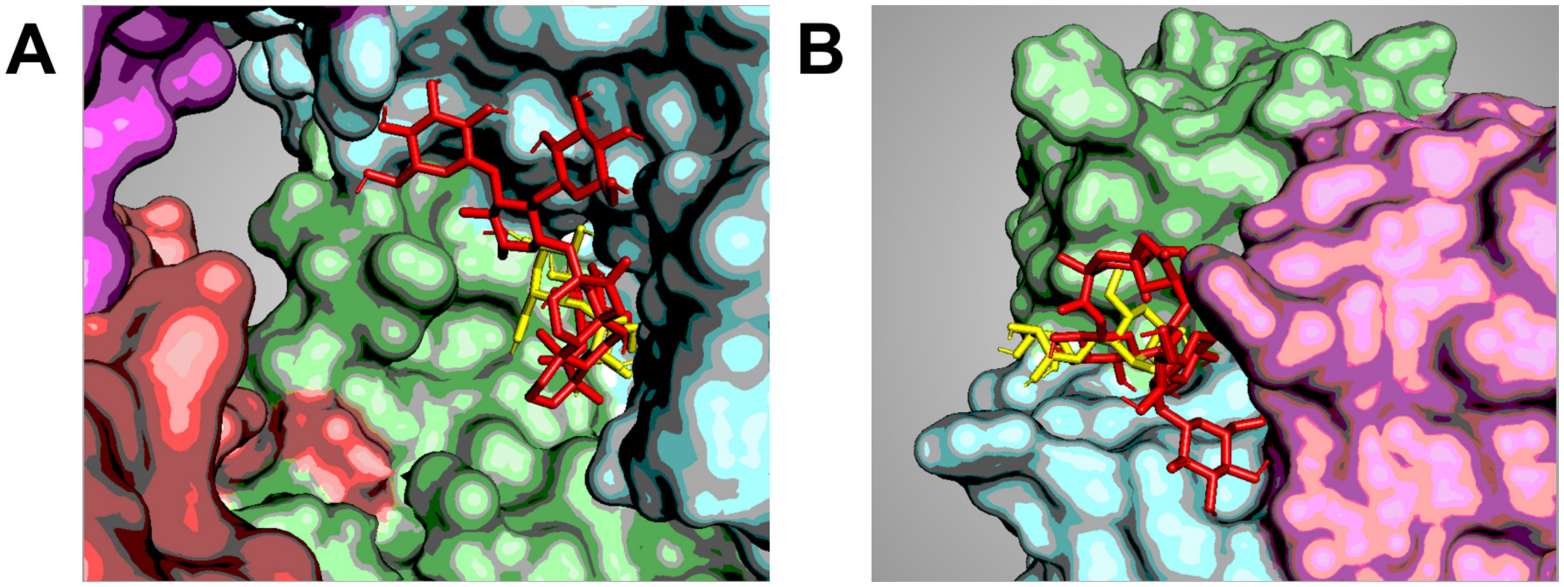
Co-localization of sweeteners on phage proteins. Cartoon showing selected poses of the docking between gp31 (a tubular protein with enzymatic activity) of Klebsiella phage 32 **(A)** and gp17 (a fiber protein involved in the recognition of host’s surface moieties) of Yersinia phage ɸYeO3-12 **(B)**. The images show the overlapping between maltose (yellow) and rebA (red) in the same pocket, suggesting a direct competition between these molecules. The images were obtained by the docking analysis carried out by Marongiu et al. (156) generated using *PyMol* ver. 2.5.0 and Schrödinger, LLC (201).

In our laboratory, we sought to assess whether this hypothesis had a foundation by investigating the binding of selected NNS on two phage proteins. We used gp31 in conjunction with the fiber protein gp17 of phage ɸYeO3-12, which has *Yersinia enterocolitica* as its host (156). We first assess the potential for NNS to bind to these proteins using biodocking. Our results indicated that several NNS could not only bind gp31 and gp17 but also overlap with the pocket binding maltose, a natural carbohydrate that represents a natural ligand for these proteins. In particular, we observed that rebA could overlap with maltose on gp31 (Figure 2A) and gp17 (Figure 2B). We confirmed the binding of rebA to recombinant gp31 by microscale thermophoresis (157) and that of gp17 by ELISA (158). Since these proteins are involved in the infection process, we sought to assess whether the binding of rebA could hamper the activity of these proteins. We observed that exposure to rebA decreased the processivity rate of gp31 compared to unexposed controls. Similarly, the addition of rebA to recombinant gp17 decreased the adsorption rate of this protein compared to controls. Unexpectedly, however, when we exposed whole phages derived from bacterial lysates, we observed that the infection process occurred about 30 min faster than in unexposed controls.

To the best of our knowledge, these experiments were the first to specifically investigate the effect of NNS on phage infectivity. These results confirmed the hypothesis that NNS (namely, rebA) could not only bind to phages but also alter the biology of these viruses. Additional experimental evidence is needed to expand these observations and understand their impact on the microbiome.

### Prophage induction

There is only one paper regarding the NNS-driven alteration of prophage induction rates (159). According to this study, stevia increased the prophage induction rate of *Bacteroides thetaiotaomicron* by 410% but decreased that of *Enterococcus fecalis*, which was instead highly induced by aspartame (+579%).

Nonetheless, carbohydrates have long been known to induce prophages with species-specific efficacy (160). For instance, glucose boosted the induction rate of *Salmonella enterica* ser. Typhimurium (161) and fructose can induce prophages Φ1 and Φ2 carried by *Limosilactobacillus reuteri*, an important commensal species of the human gut (162). Newly formed Φ1 and Φ2 virions were generated upon cultivating *L. reuteri* with either galactose, xylose, or fructose but not glucose (163). The induction mechanism was based on the reduction of fructose to mannitol, and through the action of acetate kinase A (AckA), it led to the production of the SCFA acetic acid (164). Subsequently, acetate activates the recA (163), the key regulator of the SOS response, which in turn cleaves the prophage suppressor, triggering the activation of the lytic genes (165). Interestingly, other SCFAs, such as propionate and butyrate, could induce prophage in *L. reuteri* (166). Since recA is present in virtually all bacteria and is one of the most conserved bacterial proteins (167), it is plausible that a similar induction mechanism might occur in bacteria other than *L. reuteri* (168).

## Discussion

### Summary of the data

Despite the widespread use of NNS in foodstuffs and other oral products, a consensus on the safety of these sweeteners for human consumption remains necessary. The alleged NNS food hygiene relies on the triple assumption that (i) human cells cannot metabolize these substances, (ii) they do not impact bacteria, and (iii) they reach the colon in negligible (169). Nonetheless, recent evidence suggests that NNS can cause dysbiosis in both humans and rodents, a disorder that has been linked to increased risk of conditions such as type 2 diabetes (170, 171). The purpose of this review was to provide a summary of the relationship between NNS intake and microbial activity, focusing on the experimental evidence investigating the direct effect of NNS on bacterial growth. The present work also focused on the NNS’ potential role in phage biology, a feature that is frequently overlooked in the literature.

The NNS discussed in the present review (ace-K, advantame, aspartame, neotame, saccharin, stevia, and sucralose) were consistently reported to cause dysbiosis, alter bacterial metabolism, and impair QS pathways in a species-specific fashion. These differences suggest a diverse response from selected bacterial species or even strains of the same species, which can explain the onset of dysbiosis. The data gathered in this review suggested that the primary impact of non-nutritive sweeteners (NNS) on bacterial growth is related to the induction of oxidative stress, changes in membrane permeability, and QS response. Nonetheless, it is not clear thus far whether NNS can affect the bacterial biochemistry from within after internalization or could act from outside the cell by activating signal pathways that can alter bacterial growth and environmental adaptation.

Remarkably, *in vitro* treatment of eukaryotic cells (human glioblastoma-derived SH-SY5Y and mouse cell lines TM3 and TM4) with aspartame (270 μM) or sucralose (≥1 μM) resulted in increased cellular oxidative stress (172, 173), while mouse models reported discordant results on the antioxidant effects of aspartame *in vivo* (174, 175). These results suggest that not only do NNS have the potential to affect cellular biochemistry in both prokaryotic and eukaryotic cells, but that some additional factors might superimpose on the NNS activity *in vivo*, leading to more inconsistent results.

Therefore, NNS-induced oxidative damage and cellular damage in general could be considered as the main candidates to explain the observed impairment of bacterial growth, although the details of the molecular mechanisms underlying this process are still poorly understood. Furthermore, the activation of QS pathways does not necessarily require the metabolization of NNS, which aligns with the observation that these molecules can be retrieved unaltered in biological samples (51, 176).

The possible molecular mechanisms linking NNS exposure to oxidative stress or QS alteration remain unclear. However, it is well established that oxidative stress can lead to modifications in bacterial biochemistry, including DNA damage and lipid peroxidation (177, 178). Among the bacterial responses to oxidative stress, there is the alteration of membrane fluidity (homeoviscous adaptation) and alteration of permeability through the activation of porins such as OmpC (179–181). Furthermore, oxidative stress is understood to activate the nucleotide excision repair, specifically the transcription-coupled repair pathway, which is recombinogenic (182). These responses are consistent with the results of the studies presented in the present review (53, 102).

In this review, we propose an additional putative scenario to explain NNS-linked dysbiosis: phage hindrance. The impairment of phages would not involve NNS metabolism and would most likely occur at minute levels of sweeteners due to the delicate position that phages hold in the balance of bacterial homeostasis. Such a scenario remains speculative, but so does the model of the NNS-induced QS alteration (101). The concept conveyed here is based on the observations that sucrose and other carbohydrates, most with sweetening capability, could protect the virion structure through preferential exclusion (146, 154) as well as by reducing the aggregation of viral particles (140).

In addition, recent evidence reported on NNS influence on phage infectivity (112). Because phage structural enzymes may bind saccharides such as maltose (132), it is possible that NNS could overlap with the carbohydrate-binding pocket of these proteins. In our laboratory, we have substantiated this hypothesis by showing through preliminary experiments that rebA bound proteins of phages KS32 (host: *Klebsiella pneumoniae*) and ɸYeO3-12 (host: *Yersinia enterocolitica*) and how rebA could interfere with phage infection by speeding up the lytic cycle of ɸYeO3-12 (156). The mechanisms of this enhancement, though, remain elusive.

Prophage induction might also be affected by NNS. It has been reported that stevia altered the activation of prophage in a species-specific manner (159). Since induction is linked to damage responses like the SOS pathway and QS systems (163, 183–185), the cellular stress observed in bacteria upon exposure to NNS might lead to the speculation that induction could be another by-product of NNS intake. Moreover, given the close relationship between QS and prophage induction, which is linked to horizontal gene transfer, metabolic alteration in bacteria, and predator/prey interactions (186–188), understanding the possible influence of NNS on the QS is important both for medical and microbial ecology purposes.

**Figure 3:**
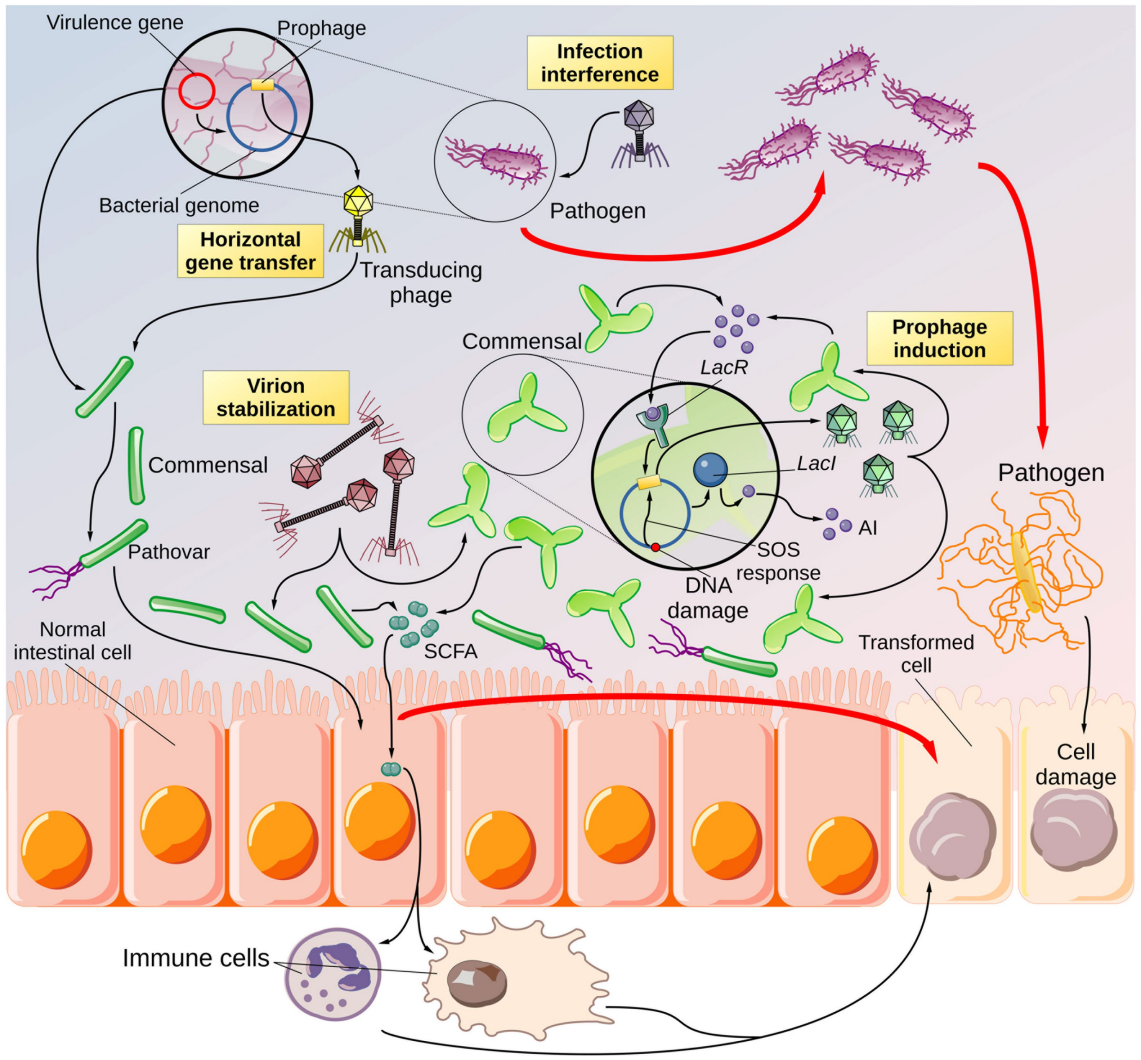
Overview of the speculative NNS-driven phage hindrance. Cartoon depicting the potential effects of NNS on phage biology and the consequences for the gut microbiota. According to the literature, NNS can increase the rate of horizontal gene transfer. It is reasonable to believe that a pathobiont (a bacterium with virulence capabilities present in the digestive system in low abundance) could exchange its virulence traits (encoded in plasmids or prophages) with a more abundant and avirulent commensal species. Thus, a commensal symbiont would be transformed into a pathovar. The virulence factors may cause harm to intestinal epithelial cells, promoting the formation of altered cells that can degenerate into cancer precursors. Inhibiting lytic phages may increase the population of pathogenic bacteria that can cause local inflammation and cellular damage if phages target pathogenic species. These conditions may, in turn, lead to the proliferation of more pathogenic species and the development of altered cells. In addition, NNS have been shown to stabilize phage virions. If the stabilized phages use a commensal species as a host, the infection rate of symbionts may increase, leading to a decrease in the abundance of beneficial species, which may promote the spread of pathogenic species. The imbalance in bacterial abundance due to phages may cause alterations in immune cells, for instance, through the action of short-chain fatty acids (SCFAs) that can lead to chronic inflammation and, consequently, cellular damage and a higher abundance of opportunistic bacteria. Finally, there are reports from the literature. NNS can influence the rate of prophage induction. Prophages can be activated in response to DNA damage, as well as to bacterial densities, due to their sensitivity to the bacterial autoinducers (AIs). Induced prophages generate a wave of infectious phages that can target other bacteria, specifically variants of the same species that do not carry the prophage, thus causing an imbalance in the bacterial community—a feature described by the community shuffling model. Even in this case, the result would be a higher risk of promoting the growth and spread of opportunistic bacteria. The end product of phage hindrance, combined with other factors such as diet and concurrent illnesses, would be the establishment of dysbiosis.

Any imbalance in bacterial abundance resulting from NNS exposure may be amplified within the microbiome, as phages influence the immune system, for example, by controlling SCFA levels (126). As a result, alterations to the immune system may produce local inflammation, which can cause cellular damage to intestinal cells and promote the spread of additional pathogens. Figure 3 illustrates this speculative framework linking the consumption of NNS to the onset of dysbiosis.

### Limitations and perspectives

Assessing NNS food hygiene is a challenging task. First, safety testing is typically performed in animal models; however, it has been noted that animal microbiomes have not proven to be a viable substitute for human intestinal microbiota (189). Second, NNS are expected to exert their activity in minute amounts over prolonged periods, making it challenging to evaluate their impact on human or bacterial cells through experimental models (50). Since most bacteria do not metabolize NNS, the mechanism underlying NNS-associated dysbiosis remains largely unknown (190). Third, studies that expose bacteria to NNS have used varying concentrations of sweeteners, complicating comparisons between results. Four, individual differences in dietary habits, genetic backgrounds, and microbiome compositions further contribute to the variability of findings across studies. Finally, it should be considered that clinical studies assessing the impact of NNS on the human microbiome are largely based on questionnaires, sometimes in the quantification of NNS levels in urine, and more rarely determine the direct impact of NNS on bacteria.

The NNS concentrations employed in the studies reported herein showed a wide variation (10^−5^-10^2^ mM) but it is not known thus far what are the physiological levels within the human GIT. To the best of our knowledge, the NNS concentration in feces has yet to be established, while only few studies reported the concentration of NNS in urine, which was in the range of 0.1–0.3% of the ADI (191). Based on an average volume of the colon of about 700 mL (192, 193), we estimated the colonic concentration of rebA at about 5 μM (Marongiu, manuscript submitted) while the environmental NNS concentration is even lower than that. Therefore, only a subset if the experiments described in the present review were performed with concentrations similar to the physiological NNS concentration.

Since alterations in bacterial growth have been observed both in the human GIT and in the environment, it can be speculated that either NNS are active at very low concentrations or some other, yet unknown, mechanisms are responsible for the reported changes. Experimental investigation, even using high NNS doses, is therefore fundamental for discriminating between these two scenarios, along with an empirical quantification of the NNS physiological level at which the bacteria are exposed within the GIT. Similarly, the data regarding the NNS effect on phages is virtually absent altogether. Additional studies addressing whether NNS could stabilize phage particles or reduce virion aggregation, for instance, would shed light on the observed alteration of phage infectivity (156).

The ramifications of NNS affecting commensal species in the intestinal tract could be far-reaching, given that these bacteria not only compete with each other for nutrient intake but also oppose the colonization of the intestinal mucosa by foreign pathogens as well as the proliferation of resident pathobionts, a process known as colonization resistance (194). For example, commensal bacteria can reduce the spread of intestinal pathogens, such as *Vibrio cholerae*, whose cholera toxin is encoded by prophage CTXΦ (195, 196), through direct competition. *Bacteroides thetaiotaomicron*, on the other hand, produces compounds that limit the growth of the pathogen *E. coli* O157: H7 as well as expression of its Shiga toxin, which is produced through the induction of Stx prophages present in this strain (197). Moreover, commensal bacteria can also counteract the invasion of pathogens by stimulating the immune system through the release of SCFA (198, 199). Thus, alterations in the growth of commensal bacteria (symbionts) might increase the susceptibility to intestinal pathogens.

Understanding the interaction between NNS and phage biology is important not only for food safety and microbial ecology but may also have direct medical applications. For instance, the addition of the sweetener xylitol to phage preparations increased the reduction in *Pseudomonas aeruginosa* and *Klebsiella pneumoniae* loads compared to phages alone (200). Nonetheless, little experimental evidence is at the moment available to fully understand how NNS might modulate phage structure and infection.

## Conclusion

The analysis of the current literature revealed a limited but growing body of evidence suggesting a connection between the consumption of NNS and dysbiosis. Despite the paucity of clinical trials, preliminary laboratory results suggest that such dysbiosis may be mediated primarily by alterations in biochemical pathways and interference with quorum sensing signaling within bacterial communities. Furthermore, NNS might influence phage biology, potentially having far-reaching consequences for the gut microbiome.
